# Systematic Review and Meta-Analysis of the Effect of Loop Diuretics on Antibiotic Pharmacokinetics

**DOI:** 10.3390/pharmaceutics15051411

**Published:** 2023-05-05

**Authors:** David A. Kerling, Sarah C. Clarke, Jesse P. DeLuca, Martin O. Evans, Adrian T. Kress, Robert J. Nadeau, Daniel J. Selig

**Affiliations:** 1 Walter Reed National Military Medical Center, Internal Medicine, Bethesda, MD 20889, USA; 2 Walter Reed Army Institute of Research, Clinical Pharmacology Fellowship, Silver Spring, MD 20910, USA

**Keywords:** antibiotics, drug interactions, loop diuretics, pharmacokinetics

## Abstract

Loop diuretics and antibiotics are commonly co-prescribed across many clinical care settings. Loop diuretics may alter antibiotic pharmacokinetics (PK) via several potential drug interactions. A systematic review of the literature was performed to investigate the impact of loop diuretics on antibiotic PK. The primary outcome metric was the ratio of means (ROM) of antibiotic PK parameters such as area under the curve (AUC) and volume of distribution (V_d_) on and off loop diuretics. Twelve crossover studies were amenable for metanalysis. Coadministration of diuretics was associated with a mean 17% increase in plasma antibiotic AUC (ROM 1.17, 95% CI 1.09–1.25, I^2^ = 0%) and a mean decrease in antibiotic V_d_ by 11% (ROM 0.89, 95% CI 0.81–0.97, I^2^ = 0%). However, the half-life was not significantly different (ROM 1.06, 95% CI 0.99–1.13, I^2^ = 26%). The remaining 13 observational and population PK studies were heterogeneous in design and population, as well as prone to bias. No large trends were collectively observed in these studies. There is currently not enough evidence to support antibiotic dosing changes based on the presence or absence of loop diuretics alone. Further studies designed and powered to detect the effect of loop diuretics on antibiotic PK are warranted in applicable patient populations.

## 1. Introduction

Diuretics are a class of medications that increase water and salt excretion and are commonly prescribed to reduce extracellular volume in edematous states [1]. Loop diuretics, such as furosemide, inhibit the sodium–potassium–chloride transporter (Na^+^–K^+^–2Cl^−^) in the thick ascending limb of the loop of Henle [2]. The result of this inhibition is increased distal excretion of sodium, chloride, and potassium.

Loop diuretics have several important physiologic effects that may alter the pharmacokinetics (PK) of antibiotics. For example, volume contraction as a result of diuretic therapy may lead to decreased volume of distribution (V_d_) of antibiotics. Regarding antibiotic clearance (CL), loop diuretics may increase urine volume, as well as the amount of antibiotic excreted in the urine [3]. However, furosemide may potentially also decrease the active secretion or increase the passive reabsorption of antibiotics. Furosemide has been shown to be an inhibitor of organic ion transporters (OAT) 1 and 3, which help to actively secrete antibiotics such as beta-lactams and cephalosporins [4,5]. Furthermore, given the disruption of the normal electrochemical gradient generated by the channel, cations such as calcium and magnesium are also excreted, creating a relatively positively charged lumen [6]. Theoretically, this could lead to paracellular reabsorption of positively charged antibiotics. Loop diuretics also may have variable effects on kidney function depending on a patient’s physiologic condition [7,8]. Hence, loop diuretics may affect the CL of renally excreted antibiotics to increase or decrease renal elimination depending on the effect of loop diuretic on the kidneys in a given patient.

Despite these potential PK interactions between loop diuretics and antibiotics, in the absence of a significant change in kidney function, it is not common for antibiotic doses to be adjusted on the basis of a patient’s diuretic regimen. As loop diuretics are a mainstay of treating volume overload and maintaining a neutral fluid balance, antibiotics are often co-prescribed. Therefore, this study aims to better understand the impact of concomitant diuretic therapy on antibiotic PK and determine if antibiotic dose adjustments may be necessary. 

## 2. Materials and Methods

### 2.1. Study Inclusion Criteria

To the authors’ knowledge, this is the first systematic review exploring the impact of loop diuretics on the pharmacokinetics of antibiotics. In addition, there are many acceptable study designs and analytic techniques to precisely estimate pharmacokinetic parameters [9]. Therefore, inclusion criteria were broad, allowing any study that reported at least one antibiotic PK parameter in patients exposed to an antibiotic and loop diuretic simultaneously. For meta-analysis, included studies were also required to include a comparison cohort that was exposed to the antibiotic alone without loop diuretic. Other types of studies were not considered for meta-analysis but were included in order to gather and describe the entire landscape of the literature. For example, single-arm cohort studies where patients only had estimates of PK parameters while concomitantly exposed to loop diuretics and antibiotics were included provided the data were adequate for exposure–response or dose–response analysis of the effect of loop diuretics on the observed antibiotic PK parameter. Articles that did not report PK parameters were still included provided the article reported time–concentration curves amenable to digitization and met the other aforementioned criteria.

Despite important physiologic differences in adult and pediatric populations, furosemide has similar physiologic effects and mechanisms of action in both populations [10]. Furthermore, after accounting for postnatal age, weight, and kidney function, the pharmacokinetics of drugs in adults and pediatrics may be reliably predicted using a single covariate PK model [11,12]. Therefore, both pediatric and adult populations were included. 

### 2.2. Search Strategy

On 16 August 2022, PubMed, Web of Science, Embase, and the Cochrane Database of Systematic reviews were searched for published manuscripts that described the effect of a loop diuretic on the pharmacokinetics of any antibiotic in humans. This review was registered in PROSPERO (CRD42022329279), and the PRISMA 2020 checklist was used as a guide to perform, complete, and report the review. A total of 1300 titles were found across the databases, with 1125 available for screening after duplicates were removed. Two investigators (D.A.K. and D.J.S.) independently screened the titles and/or abstracts. Following this stage of review, 81 articles were identified that may have met inclusion criteria, of which 77 were retrieved. Two investigators (M.O.E. and R.J.N.) independently screened the 77 full articles for inclusion, and disagreements were resolved by a single author (D.J.S.), resulting in 25 studies included in this review. The full search strategy and PRISMA 2020 flow diagram are available in the Supplementary Materials.

### 2.3. Data Collection

Data were collected by a single author (D.J.S.). Studies were categorized by design and are summarized in Table 1, Table 2 and Table 3. Data extracted from all studies were author, publication year, study design, study population, number of subjects/patients, specific loop diuretic, specific antibiotic, age, weight, and baseline estimated glomerular filtration rate (eGFR). There were five unique observational cohort studies and eight unique population PK studies. These studies were described qualitatively as analytic methods, and patient populations differed significantly amongst them. There were 12 unique crossover studies that had 16 total study arms. Given the sufficient washout time between each crossover sequence, for the purpose of analysis, different combinations of antibiotic and loop diuretics reported by a single author were considered to be independent investigations. Additional data extracted from these crossover studies included area under the curve until the last time–concentration observation (AUC_last_), volume of distribution (V_d_), half-life (T_1/2_), maximum concentration (C_max_), amount excreted in urine (A_e_), and eGFR of the antibiotic before and after loop diuretic co-administration. When available, these data were extracted manually. When not available, if time–concentration curves were reported, the data were digitized using WebPlotDigitizer (Version 4.6, Ankit Rohatgi, Pacifica, CA, USA).

### 2.4. Pharmacokinetic Analyses

Pharmacokinetic analyses were limited to the crossover studies (Table 1). The ratios of PK parameters with and without loop diuretics were the outcome metrics of interest. Some studies reported antibiotic clearance (CL), which was converted to AUC as follows:

(1) $$\begin{array}{c} \mathrm{AUC}=\frac{\mathrm{Dose}}{\mathrm{CL}}. \end{array}$$

Pharmacokinetic parameters are algebraically related. For example, V_d_ and T_1/2_ are related by the following formula:

(2) $$\begin{array}{c} T_{\frac{1}{2}}=\frac{\ln\left(2\right)\cdot V_{d}}{CL}, \end{array}$$

and V_d_ and C_max_ are related for an IV bolus dose by the following formula:

(3) $$\begin{array}{c} V_{d}=\frac{\mathrm{Dose}}{C_{0}}. \end{array}$$

Therefore, missing PK parameters such as V_d_ and T_1/2_ were not calculated from available parameters as they would be a reflection and provide similar information to other reported PK parameters. In addition, the analysis of such calculated parameters may be biased toward those reported as it relies on the reported parameters. When only time–concentration curves were available, noncompartmental analysis (NCA) was used to calculate PK parameters using the digitized data. Phoenix WinNonLin (version 8.3.5.340, Certara, Princeton, NJ, USA) was used to perform NCA.

**Table 1:** Characteristics of crossover studies.

Author	Year	Antibiotic	Diuretic	Population	N Subjects	Age (Years)	Weight (kg)	Baseline eGFR (mL/min)	Follow-Up Time
Brass et al. [13]	1974	Ampicillin 2 G IV bolus	Furosemide 40 mg PO	Healthy volunteers	8	27	75		8 h
Tice et al. [14]	1975	Cephalothin 500 mg bolus IV followed by continuous infusion 500 mg/h	Furosemide 70.5 mg IV bolus at hour 3 of continuous infusion	Healthy volunteers	5	25	70.5	133	6 h
Norrby et al. [15]	1976	Cephaloridine 1 G IM or 0.5 G IM if renal impaired	Furosemide 80 mg PO	Hospital patients	16	64.5	NA	NA	NA
Tilstone et al. ^1^ [16]	1977	Cephaloridine 250 mg IV bolus	Furosemide 20 mg IV bolus	Healthy volunteers	5	23	70	129	NA
Morgant et al. [17]	1984	Cefazolin 680 mg/h IV continuous infusion	Furosemide 20.4 mg IV bolus 1 h after start of cefazolin infusion	Healthy volunteers	6	24	68	113.4	2 h
Morgant et al. ^1^ [17]	1984	Cefazolin 680 mg/h IV continuous infusion	Piretanide 6.8 mg IV bolus 1 h after start of cefazolin infusion	Healthy volunteers	6	24	68	113.4	2 h
Chrysos et al. [18]	1995	Ceftazidime 1 G IM	Furosemide 40 mg PO 1 h prior to ceftazidime dose.	Healthy volunteers	6	NA	NA	NA	8 h
Chrysos et al. ^1^ [18]	1995	Ceftazidime 1 G IM	Furosemide 40 mg PO 3 h prior to ceftazidime dose.	Healthy volunteers	6	NA	NA	NA	8 h
Schück et al. [19]	1975	Chloramphenicol 1 G PO	Furosemide 10 mg IV bolus	Healthy volunteers	7	43.43	NA	NA	4 h
Schück et al. [20]	1978	Chloramphenicol 1 G PO	Ethacrynic acid 150 mg PO	Healthy volunteers	8	38	NA	NA	4 h
Tilstone et al. ^1^ [16]	1977	Gentamicin 20 mg IV bolus	Furosemide 20 mg IV bolus	Healthy volunteers	5	23	70	129	
Whiting et al. [3]	1981	Gentamicin 74 mg IV bolus	Furosemide 18.5 mg IV bolus	Healthy volunteers	6	27.5	74	139	7 h
Whiting et al. ^1^ [3]	1981	Gentamicin 74 mg IV bolus	Piretanide 7.4 mg IV bolus	Healthy volunteers	6	27.5	74	139	7 h
Lawson et al. [21]	1982	Gentamicin 80 mg IV bolus	Furosemide 40 mg IV bolus	Hospital patients	7	65	58	104	5 h
Hannedouche et al. [22]	1986	Gentamicin IV infusion over 30 min	Muzolimine 30 mg PO 90 min prior to gentamicin dose	Healthy volunteers	6	26.5	77	101.23	24 h
Sudoh et al. [23]	1993	Lomefloxacin 100 mg PO	Furosemide 40 mg PO	Healthy volunteers	8	30	67	NA	8 h

^1.^ These are separate study arms within the same manuscript, testing additional combinations of loop diuretics and antibiotics. NA = data not available.

**Table 2:** Characteristics of observational cohort studies.

Author	Year	Study Design	Antibiotic	Diuretic	Population	N Subjects	Age (Years)	Weight (kg)	Study Description
Adam et al. [24]	1978	Prospective cohort	Cephradine single dose infused over 20 min	Furosemide	Patients undergoing brain surgery	11	NA	NA	There were 11 patients in the cephradine group, where 6 of 11 received TID 40 mg PO furosemide 2–6 days prior to surgery with last dose 12 h prior to surgery.
Trollfors et al. [25]	1978	Prospective cohort	Cefoxitin 1 G IV infused over 30 min	Furosemide 80 mg PO	Patients with chronic infection	27	76.81	71.85	There were four study groups with antibiotic (antibiotic alone N = 7, antibiotic and daily 80 mg PO furosemide N = 12, antibiotic and 80 mg PO furosemide on days 9–11 of therapy and septic shock patients with acute renal failure). No significant changes were observed in baseline eGFR or plasma clearance of cefoxitin throughout antibiotic therapy in the no diuretic and daily diuretic groups.
Trollfors et al. [26]	1980	Prospective cohort	Cefoxitin 1–2 G IV or Cefuroxime	Furosemide 40–160 mg PO	Patients with acute or chronic infection	91	NA	NA	A total of 50 patients received cefoxitin (26 with no furosemide and 24 with furosemide), while 41 patients received cefuroxime (28 without furosemide and 13 with furosemide). No significant differences were observed in half-life of cefuroxime or cefoxitin with or without furosemide.
Marlowe et al. [27]	2003	Retrospective cohort	Vancomycin 10–25 mg/kg per dose	Furosemide	Neonates, infants and children admitted to a cardiac hospital unit	36	1.62	9.3	While not explicitly stated, the study implied that all patients were treated with furosemide. A statistically significant negative correlation between V_d_ and furosemide dose was found. However, there was no significant trend between fluid balance and V_d_, confounding the trend between V_d_ and furosemide. The effect of daily furosemide dose on vancomycin CL was not reported.
Hirai et al. [28]	2021	Retrospective cohort	Vancomycin 1000–2000 mg/day	Furosemide	Hospitalized patients	208	74	53	Furosemide alone had no statistically significant association with dose-normalized vancomycin troughs. However, a statistically significant increase in dose-normalized vancomycin trough was observed in patients receiving furosemide/thiazide diuretics combined compared to those without.

NA = not available.

**Table 3:** Characteristics of population PK studies.

Author	Year	Antibiotic	Diuretic	Population	N Subjects	N Subjects on Loop Diuretic	Age (years)	Weight	eGFR	Results of Loop Diuretic Covariate Test
Fuchs et al. [29]	2014	Gentamicin	Furosemide	Hospitalized infants	1449	5	0.65	2.17	NA	There was a non-statistically significant reduction in systemic gentamicin clearance by 34% (*p* = 0.012). There was no mention of testing on Vc. BW and age were accounted for in model.
Thibault et al. [30]	2019	Piperacillin–tazobactam	Furosemide	Hospitalized infants and children	89	25	1.5	11.4	NA	There was a statistically significant reduction (*p* < 0.05) in piperacillin CL (24%) and tazobactam CL (25%). Volume parameters were not evaluated. Weight was accounted for in model.
Lin et al. [31]	2016	Vancomycin	Furosemide	Post-craniotomy patients	100	16	51.6	59.1	104.7	Furosemide had no statistically significant effect on CL. No effect size was reported.
Medellín-Garibay et al. [32]	2016	Vancomycin	Furosemide	Hospitalized trauma patients	118	28	74.3	72	90.5	There was a statistically significant reduction (*p* < 0.05 on forward inclusion and *p* < 0.001 on backward elimination) in vancomycin CL (34%). Creatinine clearance was accounted for in model.
Medellín-Garibay et al. [33]	2017	Vancomycin	Furosemide	Patients on mechanical ventilation	54	26	65	75	106.3	Furosemide was not statistically significant; no effect size was reported.
Milovanovic et al. [34]	2019	Vancomycin	Furosemide	Patients with long bone fractures	99	23	61.12	80.32	93.23	Furosemide was not statistically significant; no effect size was reported
Xu et al. [35]	2021	Vancomycin	Furosemide	Infants with meningitis	82	26	1.13	8.27	39.24	Furosemide was not statistically significant; no effect size was reported
Buckwalter et al. [36]	2005	Dalbavancin	Furosemide	Hospitalized patients	532	79	46	88	120.6	Furosemide was not statistically significant; no effect size was reported.

NA = not available.

### 2.5. Meta-Analysis and Risk of Bias Assessment

The ratio of mean PK parameters is a standard and informative way to present the effect of a covariate on PK parameters or the bioequivalence of a new formulation of a product compared to its reference product [37,38]. Therefore, the outcome measure chosen was the ratio of mean PK parameters with and without a loop diuretic. The outcome measure was calculated using the “escalc” function in the metafor package (version 3.8) using R (version 4.2, R Foundation for Statistical Computing, Vienna, Austria) and R Studio (version 2022.07.2+576, RStudio Team, Boston, MA, USA) [39]. When the standard deviation of PK parameters was not reported, or when generating mean parameters such as V_d_ using Equation (2), variability was assumed to be 20% CV, which was generally consistent with the data reported in the crossover studies. The meta-analyses and forest plots were performed using the “metagen” and “forest.meta” functions, respectively, from the meta package (version 6.0), using a random effects model with the meta default presets and summary measure set to “ROM” for ratio of means [40]. 

In the crossover studies amenable for meta-analysis, no clear patterns emerged to elucidate heterogeneity. Aside from two studies in hospitalized patients, demographic variables were fairly uniform, spanning a relatively short range. Older age and lower weight found in hospitalized patients were confounded by hospital status, in addition to a higher risk of bias in studies in hospitalized patients due to a higher likelihood of carryover effects. Given these limitations, heterogeneity was primarily explored qualitatively and is described in the discussion. However, data were sufficient to explore subgroup analysis by antibiotic class and specific loop diuretic (furosemide vs. other diuretic). Missing data were excluded from the meta-analysis. No sensitivity analyses were performed because the nature of this meta-analysis was exploratory, and only very large effects on PK parameters require clinical dose changes [41].

The risk of bias was assessed collaboratively by two authors (A.T.K. and J.P.D.) using the revised Cochrane risk-of-bias tool for randomized crossover trials (RoB 2) [42]. The risk of bias was visualized using the robvis visualization tool [43]. Although the RoB 2 tool was used as a guide, the decision tree did not align perfectly with PK studies, which may be less prone to bias. This is elaborated in the discussion.

## 3. Results

### 3.1. Crossover Studies

There were 12 unique crossover studies with 16 total study arms including 88 subjects (Table 1). The most common study design (N = 9 studies) was a two-sequence crossover (antibiotic and loop diuretic + antibiotic) after an appropriate washout period [3,13,16,18,19,20,21,22,23]. Two of these studies tested multiple antibiotics or diuretics [3,43], and one study tested different timing of loop diuretic dosing prior to the antibiotic [16]. Protocols for sequence allocation and randomization were inconsistently reported. Two studies employed the use of a continuous antibiotic infusion, providing a bolus dose of loop diuretic after achieving a steady state [14,17]. Norrby et al. utilized a three-sequence crossover design in hospitalized patients (antibiotic, antibiotic + loop diuretic, and antibiotic) [15]. Most commonly, crossover studies were performed in healthy adults (N = 10 studies). The remaining two studies were performed in hospitalized patients [20]. The median and ranges for age, weight, and eGFR were 27.5 years (23–65), 70.25 kg (58–77), and 121.2 mL/min (101.23–139), which were reported in 11, eight, and six studies, respectively.

**Figure 1:**
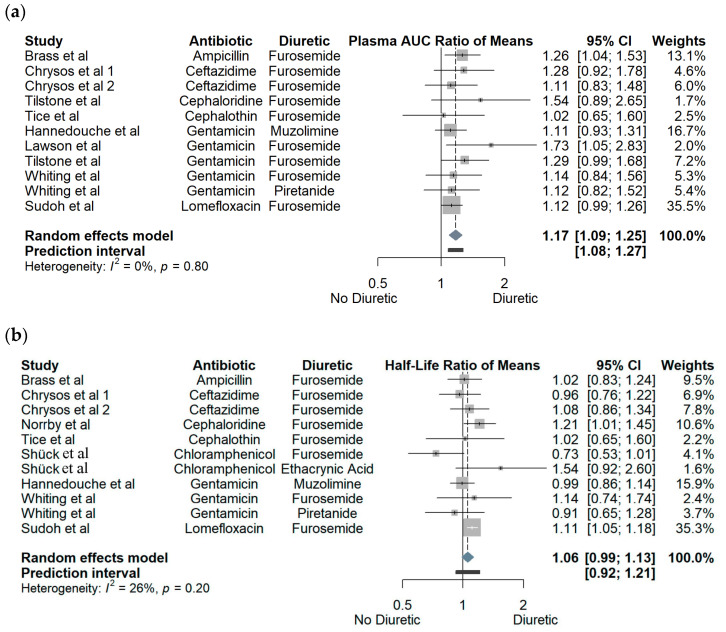

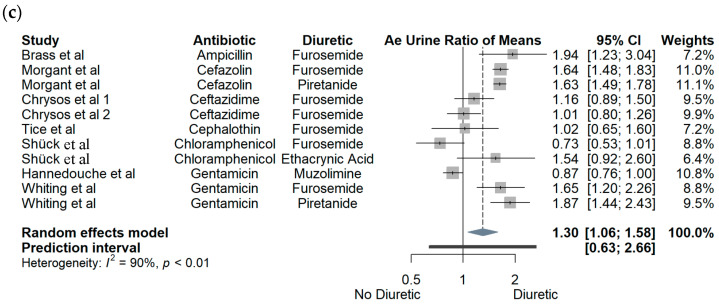
Forest plots of effect of loop diuretic on (**a**) area under the curve (AUC); (**b**) half-life (T_1/2_); (**c**) amount of drug in urine (A_e_). Chrysos et al 1 (furosemide dosed 1 h prior to antibiotic) and Chrysos et al 2 (furosemide dosed 3 h prior to antibiotic) refer to two separate study arms within the Chrysos et al. study [3,13,14,15,16,17,18,19,20,21,22,23].

**Figure 2:**
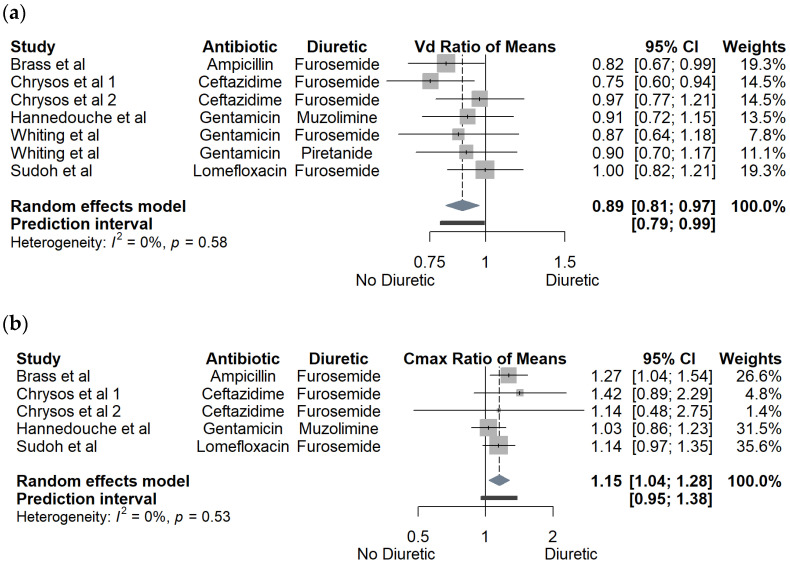

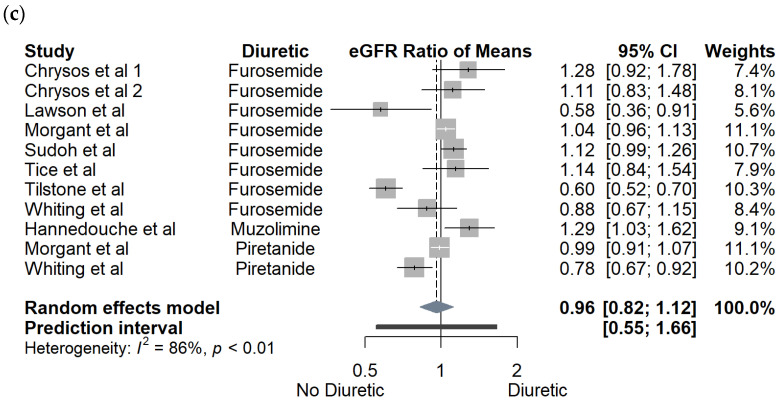
Forest plots of effect of loop diuretic on (**a**) volume of distribution (V_d_); (**b**) maximum concentration (C_max_); (**c**) estimated glomerular filtration rate (eGFR). Chrysos et al 1 (furosemide dosed 1 h prior to antibiotic) and Chrysos et al 2 (furosemide dosed 3 h prior to antibiotic) refer to two separate study arms within the Chrysos et al study [3,13,14,16,17,18,21,22,23].

Pooled effect estimates of loop diuretics on PK parameters AUC, T_1/2_, V_d_, C_max_, A_e_, and eGFR are summarized in Figure 1a–c and Figure 2a–c. Subgroup analysis by antibiotic category or by type of loop diuretic did not reveal any statistically significant or clinically relevant differences. Findings of subgroup analyses are summarized in Tables S1 and S2 in the Supplementary Materials. Presence of a loop diuretic was associated with an overall 17% (2–54%) increase in plasma AUC. The direction of all effect estimates for AUC was homogeneous, regardless of antibiotic or loop diuretic. Although only two studies individually achieved statistical significance, the overall mean effect was statistically significant with a confidence interval of 9–25% increase in plasma AUC. Similarly, the presence of a loop diuretic was associated with an overall statistically significant mean decrease of 11% for V_d_, with 0% heterogeneity; however, again, only two individual studies achieved statistical significance. Half-life was largely unchanged in the presence of a loop diuretic, with no study demonstrating a significant effect, and with a mean non-statistically significant 6% increase. Given that AUC increased and V_d_ decreased in similar proportions, the finding of a nearly unchanged half-life is expected as T_1/2_ is mathematically determined by the ratio of CL and V_d_, and CL is inversely proportional to AUC (see Equations (1) and (2)). An observed overall mean increase of 15% in C_max_ was consistent with the observed decrease in V_d_, as C_max_ and V_d_ are inversely proportional (see Equation (3)). Presence of a loop diuretic was associated with a statistically significant 30% increase in A_e_; however, heterogeneity was 90%, with a large prediction interval crossing one. There were insufficient data for subgroup analysis; however, there was no obvious trend suggesting that an antibiotic class or specific diuretic would explain the heterogeneity. Generally, presence of a loop diuretic was associated with a minimal effect on eGFR, i.e., an overall nonsignificant mean effect of a 4% reduction. However, Lawson et al. [21] and Tilstone et al. [16] demonstrated remarkably large mean eGFR reductions of 42% and 40%, respectively, associated with the presence of a loop diuretic.

**Figure 3:**
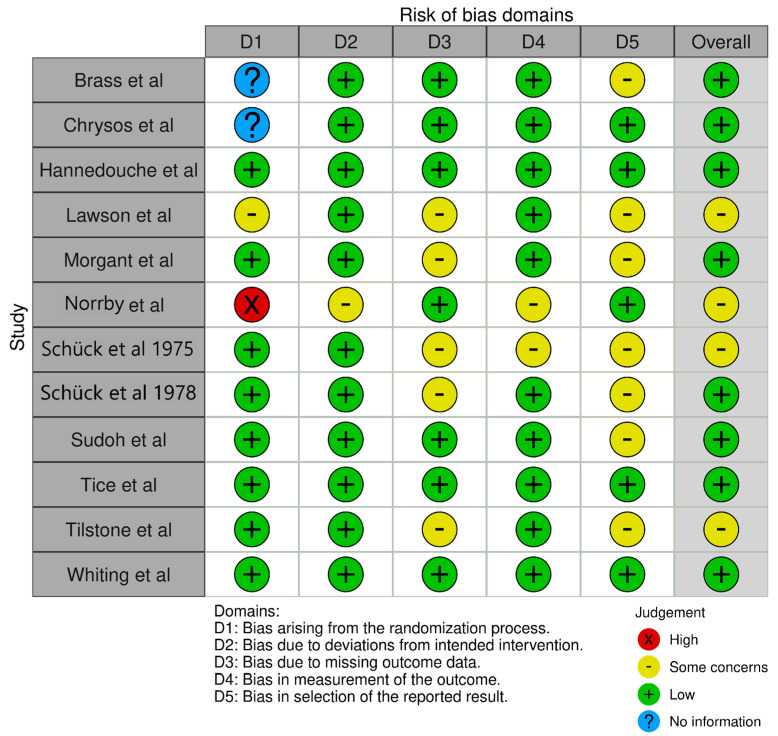
Risk-of-bias plot for crossover studies [3,13,14,15,16,17,18,19,20,21,22,23].

The risk of bias for crossover studies is summarized in Figure 3. In order to utilize the tool with pharmacokinetic studies, we had to maintain some flexibility in how we applied the domains, as not all of them were clearly applicable to these types of studies. Overall, eight of 12 crossover studies were assessed to have a low risk of bias, with the other four having a modest risk of bias. Most of the studies were performed on young, healthy volunteers, whereas Norrby et al. [15] and Lawson et al. [21] studied older patients with serious medical conditions requiring hospitalization. The main areas of modest concern for bias fell in domains 3 and 5; primarily related to the possibility of providing patient-specific information instead of only pooled data. Norrby et al. had an unequal allocation of patients to sequences (two vs. 14) and higher potential for carryover effects and other confounding variables, given that patients were hospitalized [15]. 

### 3.2. Observational Cohort Studies

The five observational cohort studies included in this review are summarized in Table 2. In three studies, data were collected prospectively [24,25,26], whereas, in two studies, data were collected retrospectively [27,28]. Patient populations differed significantly within each study, ranging from neonates to adults scheduled for brain surgery. There were a total of 373 patients across these studies, with Hirai et al. contributing 55.8% (N = 208). Analytic techniques and primary outcomes were different among the studies. Adam et al. performed statistical comparisons of individual time–concentration relationships and found that serum concentrations were statistically higher in the furosemide + antibiotic group compared to the antibiotic group alone [24]. Trollfors et al., in one study, demonstrated no significant change in antibiotic CL or V_d_ from baseline day 1 to days 12–16 in the antibiotic vs. antibiotic + loop diuretic cohorts [44]. Neither Adam et al. nor Trollfors et al. clearly stated whether patients were randomized, which makes these studies susceptible to bias, given the large range of physiologic and hospital factors that may affect PK over time [45]. These studies did not explore other covariate effects on PK. A second Trollfors et al. study demonstrated no significant difference in antibiotic half-life in patients receiving antibiotic or cefoxitin and a loop diuretic across a large range of eGFR. This Trollfors et al. study performed linear regression and accounted for eGFR in the model; however, clinically irrelevant differences in cefoxitin T_1/2_ were still found between the groups. Marlowe et al. performed a pediatric PK study, collecting antibiotic vancomycin samples in 36 patients and performing a linear regression analysis to explore the effect of total diuretic dose on PK parameters [27]. Although Marlowe et al. concluded that there was a significant negative correlation between total furosemide dose and V_d_, there was no significant trend between total fluid balance and V_d_. The reason for this is unclear and suggests that the effect of furosemide on vancomycin V_d_ in this study may have been biased. Hirai et al. [28] demonstrated through multiple linear regression analyses that the combination of a loop/thiazide diuretic was associated with a statistically significant increase in vancomycin trough (+4.47 mg/L). However, this finding was only at the initial dosing period and did not remain statistically significant when exploring the final or mean vancomycin troughs; moreover, it was not significant with loop diuretics alone. Overall, these studies were very heterogeneous in design, analysis, and outcome, as well as highly prone to bias. However, in general, these studies suggested a potentially slight increase in serum antibiotic levels associated with loop diuretic dosing, although this increase is not likely to be clinically significant. 

### 3.3. Population Pharmacokinetic Studies

Included in this review were eight population PK studies gathering data from a total of 2523 patients with an average of 9% (N = 228) receiving a loop diuretic [29,30,31,32,33,34,35,36]. The median and range of age, weight, and eGFR across the studies were 26.65 years (0.65–65), 65.55 kg (2.17–88), and 98.97 mL/min (39.24–120.6), respectively. These studies had an observational cohort design and were not specifically designed or powered to test the effect of loop diuretics on PK parameters. Rather, they were designed to explore PK and covariates in special populations such as hospitalized children and infants or patients on mechanical ventilation [30,33]. The number of patients in each study on loop diuretics was generally obtained through a convenience sample and varied greatly. For example, Fuchs et al. only had PK data on less than 1% (N = 5) of patients on loop diuretics, while Medellín-Garibay et al. had almost 50% of patients on a loop diuretic (N = 26). There were many potential confounders of PK estimates such as differing weight, eGFR, concomitant medications, and other factors attributable to the specific setting of care. Nevertheless, the development of population PK covariate models is an intensive process that can reliably account for multiple covariate effects and provide precise estimates for PK parameters, as well as quantify covariate effects [46]. Therefore, although there is a strong possibility of a biased covariate effect estimate for loop diuretics in any individual study, these population PK studies were included and classified separately from other observational cohort studies. Only two studies reported a statistically significant covariate effect of loop diuretics on antibiotic CL, reporting effect size estimates of 25% and 34% reductions in antibiotic CL. A third study reported a non-statistically significant trend of 34% reduced antibiotic clearance in association with concomitant loop diuretic prescription. The remaining five studies found that loop diuretics had no statistically significant effect on antibiotic clearance but did not report estimated effect sizes. No study clearly reported the timing of loop diuretic dosing in relation to the antibiotic dose, and no study explored the effect of loop diuretics on PK parameters over time. Overall, loop diuretics in these studies did not have a statistically or clinically significant effect on CL that allowed recommending dose changes. Interestingly, of the three studies that reported effect size, all demonstrated a reduction in antibiotic CL, which is consistent with the observed increased plasma AUC and plasma concentrations reported in the crossover and observational studies described above.

## 4. Discussion

To our knowledge, this is the first systematic review and meta-analysis to assess the impact of loop diuretics on antibiotic pharmacokinetics. In crossover studies, we found that loop diuretics were consistently associated with moderate increases in antibiotic plasma AUC, decreases in antibiotic V_d_, and minimal changes in antibiotic T_1/2_. Interestingly, there was also a trend toward a significantly increased antibiotic A_e_ of 30% (ROM 1.3, 95% CI 1.06–1.58). The observed increases in AUC and Ae may be partially explained by volume contraction. However, V_d_ does not mathematically affect AUC, and the decrease in V_d_ was only 11% on average, compared to a 30% increase in A_e_ on average. Therefore, we hypothesize that another physiologic process must also account for the increased AUC. One possible mechanism may be the reduction in active antibiotic secretion via OAT1 and OAT3 as a result of furosemide transporter inhibition. The observed increase in AUC is less likely explained by electrostatic interactions leading to greater passive reabsorption. Cephalosporins are generally known to be negatively or neutrally charged at physiologic pH [47], whereas aminoglycosides may be neutrally or positively charged [48,49]. However, increased AUC was observed for all antibiotics regardless of class.

Although the increase in AUC associated with loop diuretic use in the crossover studies was modest (mean 17%), no standard clinical antibiotic dose adjustments are recommended on the basis of this finding. The crossover studies mostly included healthy adults, greatly limiting the ability to extrapolate results to more common clinical scenarios. Furthermore, a 17% increase in AUC is not necessarily a large enough effect size to warrant standard antibiotic dose decreases, and there were insufficient data on repeat concomitant dosing to understand the PK of repeatedly dosed antibiotics with loop diuretics. Therefore, even for a generally healthy patient where the crossovers studies may apply, a standard antibiotic dose reduction is not warranted. For example, consider a case where an adult with normal kidney function is taking chronic loop diuretic therapy for venous insufficiency. If this patient developed a mild community-acquired pneumonia and required amoxicillin–clavulanate therapy, a 17% dose decrease used to account for the possible increase in AUC would result in a prescription of approximately 725 mg amoxicillin compared to 825 mg amoxicillin twice daily. Amoxicillin has a large therapeutic window, with mostly idiosyncratic adverse effects [50]. Therefore, the possible harm due to treatment failure with dose reduction would not outweigh the possible benefit of reduced exposure. 

The observational cohort studies and population PK studies were very heterogeneous in design, population, and outcome measure. Of note, three of eight population PK studies found furosemide to have a statistically significant effect on antibiotic CL, with a reduction of 20–30%. However, many covariates are often tested when developing population PK models. This raises the possibility of a false positive due to multiplicity, especially given various other factors that could alter antibiotic PK in a hospital setting [51,52]. Furthermore, these studies were not designed to detect a difference in antibiotic PK due to furosemide, as only one study had five of 1449 patients receive a loop diuretic. In addition, neither the remaining five population PK studies nor the five observational cohort studies found a significant reduction in antibiotic CL. Therefore, in a general hospital or critical care setting, there is not enough evidence to recommend standard antibiotic dose changes when co-prescribing with loop diuretics.

Further limitations of the results of this study include limited data on specific antibiotics and wide variations in how the loop diuretic was administered. For example, some authors tested the effect of loop diuretics administered orally 1–3 h prior to antibiotic administration, while others tested co-administration of intravenous loop diuretics with an antibiotic. The timing of loop diuretic dosing in relation to antibiotic dosing in the cohort studies and population PK studies was not commonly reported. Furosemide is known for highly variable oral bioavailability, especially in edematous states [53]. Since the half-life is approximately 2 h, dosing furosemide orally vs. intravenously at different times prior to antibiotic administration may have a large effect on the potential diuretic–antibiotic interaction. This phenomenon was hinted at by Chrysos et al. [18], where administration of furosemide 1 h prior to antibiotic was associated with a 28% increase in antibiotic AUC, while furosemide administered 3 h prior to antibiotic was associated with an 11% increase in antibiotic AUC (Chrysos et al. 1 and Chrysos et al. 2 in the forest plots, respectively).

## 5. Conclusions

Overall, there appears to be a consistently observed increase in AUC for antibiotics associated with loop diuretic coadministration in healthy adults. This result should not be extrapolated to patient populations commonly receiving concomitant prescriptions of antibiotics and loop diuretics. There was not strong enough evidence from the observational cohort studies or population PK studies to recommend antibiotic dosing changes in corresponding patient populations. However, there remains a potential for loop diuretics to impact antibiotic PK, and further research is warranted to better characterize antibiotic PK when concomitantly prescribing a loop diuretic in applicable patient populations. Loop diuretics may alter antibiotic PK via different mechanisms depending on the underlying physiology. Further research should focus on specific patient populations that are narrowly defined to better isolate the effect of loop diuretics on antibiotic PK.

## Data Availability

Data are available upon reasonable request.

## Supplementary Materials

The following supporting information can be downloaded at https://www.mdpi.com/article/10.3390/pharmaceutics15051411/s1. Supplementary S1: PRISMA flow diagram and full search strategy; Supplementary S2: PRISMA checklist; Tables S1 and S2: Summary of subgroup analysis. Reference [54] is cited in Supplementary Materials.
