# Supplementary material for: Systematic Review and Meta-Analysis of the Effect of Loop Diuretics on Antibiotic Pharmacokinetics
Source: Pharmaceutics. 2023 May 5;15(5):1411. doi: 10.3390/pharmaceutics15051411 (PMC10224453; doi:10.3390/pharmaceutics15051411)
Supplement: Supplementary file 1 [file pharmaceutics-15-01411-s001.zip › Supplementary S1.pdf]

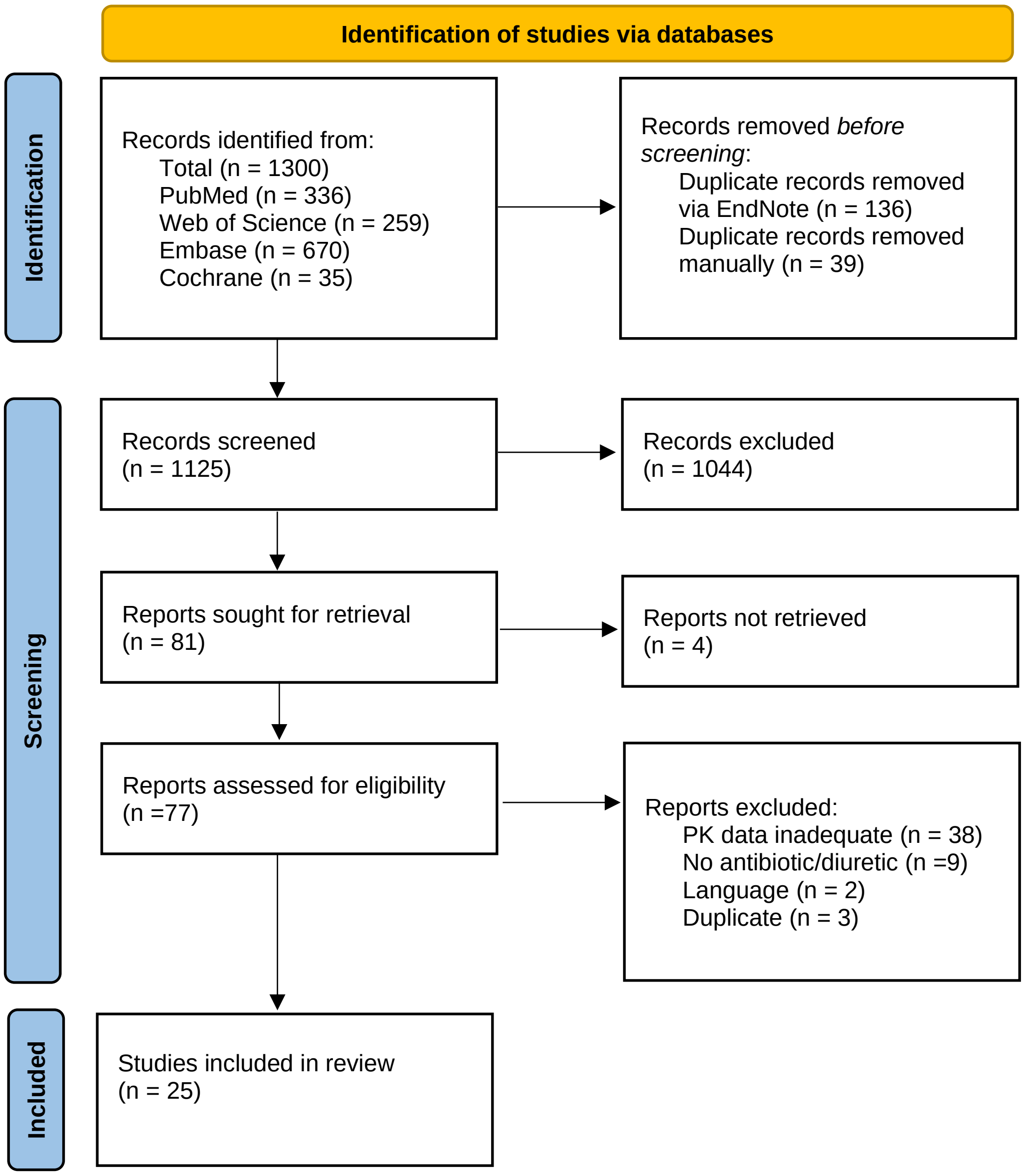

From: Page MJ, McKenzie JE, Bossuyt PM, Boutron I, Hoffmann TC, Mulrow CD, et al. The PRISMA 2020 statement: an updated guideline for reporting systematic reviews. BMJ 2021;372:n71. doi: 10.1136/bmj.n71

For more information, visit: http://www.prisma-statement.org/

PubMed

336 results on 16 August 2022

(((("bumetanide"[mesh] OR "bumetanide"[tiab] OR "bumethanide"[tiab] OR "bumex"[tiab] OR "burinex"[tiab] OR "demadex"[tiab] OR "edecrin"[tiab] OR "errolon"[tiab] OR "etacrynic acid"[tiab] OR "ethacrinic acid"[tiab] OR "ethacrynate sodium"[tiab] OR "ethacrynic acid"[mesh] OR "ethacrynic acid"[tiab] OR "fordiuran"[tiab] OR "frusemid"[tiab] OR "frusemide"[tiab] OR "furanthril"[tiab] OR "furosemide"[mesh] OR "furosemide"[tiab] OR "fusid"[tiab] OR "high ceiling diuretics"[tiab] OR "hydromedin"[tiab] OR "lasix"[tiab] OR "loop diuretic"[tiab] OR "loop diuretics"[tiab] OR "loop inhibitor"[tiab] OR "loop inhibitors"[tiab] OR "piretanide"[tiab] OR "sodium potassium chloride symporter inhibitors"[mesh] OR "sodium potassium chloride symporter inhibitors"[pharmacological action] OR "sodium potassium chloride symporter inhibitors"[tiab] OR "torasemide"[tiab] OR "torsemide"[mesh])) AND ("aminoglycoside"[tiab] OR "aminoglycosides"[mesh] OR "amoxicillin"[tiab] OR "amphenicol"[tiab] OR "amphenicols"[tiab] OR "ampicillin"[tiab] OR "anti bacterial agent"[tiab] OR "anti bacterial agents"[tiab] OR "anti bacterial compound"[tiab] OR "anti bacterial compounds"[tiab] OR "anti mycobacterial agent"[tiab] OR "anti mycobacterial agents"[tiab] OR "anti-bacteria"[tiab] OR "antibacterial agent"[tiab] OR "anti-bacterial agent"[tiab] OR "anti-bacterial agents"[mesh] OR "anti-bacterial agents"[pharmacological action] OR "antibacterial agents"[tiab] OR "anti-bacterial agents"[tiab] OR "anti-bacterial compound"[tiab] OR "anti-bacterial compounds"[tiab] OR "antibiotic"[tiab] OR "anti-biotic"[tiab] OR "antibiotics"[tiab] OR "anti-biotics"[tiab] OR "antimycobacterial agent"[tiab] OR "anti-mycobacterial agent"[tiab] OR "antimycobacterial agents"[tiab] OR "anti-mycobacterial agents"[tiab] OR "azithromycin"[tiab] OR "bacteriocidal agent"[tiab] OR "bacteriocidal agents"[tiab] OR "bacteriocide"[tiab] OR "bacteriocides"[tiab] OR "beta-lactam"[tiab] OR "cefaclor"[tiab] OR "cefadroxil"[tiab] OR "cefazolin"[tiab] OR "cefixime"[tiab] OR "cefoxitin"[tiab] OR "ceftriaxone"[tiab] OR "cefuroxime"[tiab] OR "cephalexin"[tiab] OR "cephalosporanic acid"[tiab] OR "cephalosporanic acids"[tiab] OR "cephalosporin antibiotic"[tiab] OR "cephalosporin antibiotics"[tiab] OR "cephalosporin"[tiab] OR "cephalosporins"[mesh] OR "chloramphenicol"[mesh] OR "chlornitromycin"[tiab] OR "chlorocid"[tiab] OR "chloromycetin"[tiab] OR "ciprofloxacin"[tiab] OR "clarithromycin"[tiab] OR "cloranfenicol"[tiab] OR "dalbavancin"[tiab] OR "detreomycin"[tiab] OR "dicloxacillin"[tiab] OR "doxycycline"[tiab] OR "erythromycin"[tiab] OR "fidaxomicin"[tiab] OR "fluoroquinolone"[tiab] OR "fluoroquinolones"[mesh] OR "fluoroquinolones"[tiab] OR "fucithalmic"[tiab] OR "furadantin"[tiab] OR "furadantine"[tiab] OR "furadoine"[tiab] OR "furadonine"[tiab] OR "furantoin"[tiab] OR "fusidate sodium"[tiab] OR "fusidic acid"[mesh] OR "fusidic acid"[tiab] OR "fusidin"[tiab] OR "gentamicin"[tiab] OR "levofloxacin"[tiab] OR "levomycetin"[tiab] OR "macrodantin"[tiab] OR "macrolide"[tiab] OR "macrolides"[mesh] OR "minocycline"[tiab] OR "moxifloxacin"[tiab] OR "nafcillin"[tiab] OR "nitrofurantoin"[mesh] OR "ofloxacin"[tiab] OR "ophthochlor"[tiab] OR "oritavancin"[tiab] OR "oxacillin"[tiab] OR "penicillin g"[tiab] OR "penicillin v"[tiab] OR "penicillin"[tiab] OR "penicillin"[tiab] OR "penicillins"[mesh] OR "phenoxymethylpenicillin"[tiab] OR "proloprim"[tiab] OR "roxithromycin"[tiab] OR "sarecycline"[tiab] OR "silver fusidate"[tiab] OR "sodium fusidate"[tiab] OR "stanicide"[tiab] OR "sulfacetamide"[tiab] OR "sulfadiazine silver"[tiab] OR "sulfasalazine"[tiab] OR "syntomycin"[tiab] OR "telavancin"[tiab] OR "tetracyclines"[mesh] OR "trimethoprim"[mesh] OR "trimpex"[tiab] OR "vancocin hcl"[tiab] OR "vancocin"[tiab] OR "vancocine"[tiab] OR "vancomycin hydrochloride"[tiab] OR "vancomycin sulfate"[tiab] OR "vancomycin"[mesh] OR "vancomycin"[tiab])) AND ("gfr"[tiab] OR "glomerular filtration rate"[mesh] OR "glomerular filtration rate"[tiab] OR "glomerular filtration rates"[tiab] OR "inulin clearance"[tiab] OR "kidney function test"[tiab] OR "kidney function tests"[mesh] OR "kidney function tests"[tiab] OR "kidney function"[tiab] OR metabolism[MeSH] OR "Pharmacogenomic Variants"[Mesh] OR "Pharmacokinetics"[Mesh] OR "pharmacokinetics"[Subheading] OR "metabolic clearance rate"[mesh] OR "clearance rate"[tiab] OR "clearance rates"[tiab] OR "renal clearance"[tiab] OR "renal function"[tiab] OR metaboli*[tiab] OR Pharmacokinetic*[tiab])) NOT ("animals"[mesh] NOT "humans"[mesh])

Full WOS: Web of Science Core Collection [Science Citation Index Expanded (SCI-EXPANDED) 1996-present, Social Sciences Citation Index (SSCI) 1996-present), Arts & Humanities Citation Index (AHCI) 1996-present, Conference Proceedings Citation Index – Science (CPCI-S) 1996-present, Conference Proceedinjgs Index Social Sciences & Humanities (CPCI-SSH) 1996-present), Book Citation Index – Science (BKCI-S) 2005-present, Book Citation Index –

Social Sciences & Humanities (BKCI-SSH) 2005-present, Emerging Sources Citation Index (ESCI) 2017-present, Current Chemical Reactions (CCR-EXPANDED) 1985-present, Index Chemicus (IC) 1993-present], BIOSIS Citation Index, Current Contents Connect, Data Citation Index, KCI-Korean Journal Database, MEDLINE, SciELO Citation Index

259 results on 16 August 2022

TS=("etacrynic acid" OR "ethacrinic acid" OR "ethacrynate sodium" OR "ethacrynic acid" OR "high ceiling diuretics" OR "loop diuretic" OR "loop diuretics" OR "loop inhibitor" OR "loop inhibitors" OR "pirentanide" OR "sodium potassium chloride symporter inhibitors" OR "torasemide" OR bumetanide OR bumetanide OR bomex OR burined OR demodex OR edecin OR erroron OR fordtran OR fursemide OR fursemide OR furantril OR furosemide OR fused OR hydromedion OR Lasix) AND TS=("antibacterial agent" OR "anti-bacterial agent" OR "antibacterial agents" OR "anti-bacterial agents" OR "antibacterial compound" OR "anti-bacterial compound" OR "antibacterial compounds" OR "anti-bacterial compounds" OR "antimycobacterial agent" OR "anti-mycobacterial agent" OR "antimycobacterial agents" OR "anti-mycobacterial agents" OR "bacteriocidal agent" OR "bacteriocidal agents" OR "cephalosporanic acid" OR "cephalosporanic acids" OR "cephalosporin antibiotic" OR "cephalosporin antibiotics" OR "fusidate sodium" OR "fusidic acid" OR "penicillin g" OR "penicillin v" OR "silver fusidate" OR "sodium fusidate" OR "sulfadiazine silver" OR "vancocin hcl" OR "vancomycin hydrochloride" OR "vancomycin sulfate" OR aminoglycoside* OR amoxicillin OR amphenicol OR amphenicol OR ampicillin OR anti-bacteria* OR antibacterial* OR antibiotic* OR anti-biotic* OR azithromycin OR bacteriocide* OR beta-lactam OR cefaclor OR cefadroxil OR cefazolin OR cefixime OR cefoxitin OR ceftriaxone OR cefuroxime OR cephalexin OR cephalosporin OR cephalosporins OR chloramphenicol OR chloronitromycin OR chloropid OR chloromycetin OR ciprofloxacin OR clarithromycin OR chloramfenicol OR dalbavancin OR dextromycin OR dicloxacillin OR doxycycline OR erythromycin OR fidaxomicin OR fluoroquinolone* OR fucitalmic OR furadantin* OR furadonina OR furadonina OR furantoin OR fusidic OR gentamicin OR levofloxacin OR levomycetin OR macrolactin OR macrolide* OR minocycline OR moxifloxacin OR nafcillin OR nitrofurantoin OR ofloxacin OR ochthochloa OR oritavancin OR oxacillin OR penicillin* OR phenoxymethylpenicillin OR poloprism OR roxithromycin OR samacycline OR staticide OR sulfacetamide OR sulfasalazine OR synthomycin OR telavancin OR tetracyclines OR trimethoprim OR trimper OR vancocin OR vancocin OR vancomycin) AND TS=("glomerular filtration rate" OR "glomerular filtration rates" OR "inulin clearance" OR "kidney function test" OR "kidney function tests" OR "kidney function" OR "Pharmacogenomic Variants" OR "renal clearance" OR "renal function" OR gfr OR ((rate* OR clearance) AND (metaboli* OR Pharmacokinetic*)) OR "clearance rate" OR "clearance rates") NOT (TS=animal* NOT TS=human*)

Embase

670 results on 16 August 2022

('bumetanide'/exp OR 'loop diuretic agent'/exp OR 'ethacrynic acid'/exp OR 'furosemide'/exp OR 'sodium potassium chloride symporter inhibitors'/exp OR 'torsemide'/exp OR bumetanide:ab,ti OR bumethanide:ab,ti OR bumex:ab,ti OR burinex:ab,ti OR demadex:ab,ti OR edecrin:ab,ti OR errolon:ab,ti OR 'etacrynic acid':ab,ti OR 'ethacrinic acid':ab,ti OR 'ethacrynate sodium':ab,ti OR 'ethacrynic acid':ab,ti OR fordiuran:ab,ti OR frusemid:ab,ti OR frusemide:ab,ti OR furanthril:ab,ti OR furosemide:ab,ti OR fusid:ab,ti OR 'high ceiling diuretics':ab,ti OR hydromedin:ab,ti OR lasix:ab,ti OR 'loop diuretic':ab,ti OR 'loop diuretics':ab,ti OR 'loop inhibitor':ab,ti OR 'loop inhibitors':ab,ti OR piretanide:ab,ti OR 'sodium potassium chloride symporter inhibitors':ab,ti OR torasemide:ab,ti) AND ('fusidic acid'/exp OR 'aminoglycoside'/exp OR 'cephalosporin derivative'/exp OR 'chloramphenicol'/exp OR 'quinolone derivative'/exp OR 'macrolide'/exp OR 'nitrofurantoin'/exp OR 'penicillin derivative'/exp OR 'tetracycline derivative'/exp OR 'trimethoprim'/exp OR 'vancomycin'/exp OR 'aminoglycoside':ti OR 'amoxicillin':ti OR 'amphenicol':ab,ti OR 'amphenicols':ti OR 'ampicillin':ti OR 'anti bacterial agent':ti OR 'anti bacterial agents':ti OR 'anti bacterial compound':ti OR 'anti bacterial compounds':ti OR 'anti mycobacterial agent':ti OR 'anti mycobacterial agents':ti OR 'anti-bacteria':ti OR 'antibacterial agent':ti OR 'anti-bacterial agent':ti OR 'antibacterial agents':ti OR 'anti-bacterial agents':ti OR 'anti-bacterial compound':ti OR 'anti-bacterial compounds':ti OR 'antibiotic':ti OR 'anti-biotic':ti OR 'antibiotics':ti OR 'anti-biotics':ti OR 'antimycobacterial agent':ti OR 'anti-mycobacterial agent':ti OR 'antimycobacterial agents':ti OR 'anti-mycobacterial agents':ti OR 'azithromycin':ab,ti OR 'bacteriocidal agent':ti OR 'bacteriocidal agents':ti OR 'bacteriocide':ti OR 'bacteriocides':ti OR 'beta-lactam':ti OR 'cefaclor':ti OR 'cefadroxil':ti OR 'cefazolin':ti OR 'cefixime':ti OR 'cefoxitin':ti OR 'ceftriaxone':ti OR 'cefuroxime':ti OR 'cephalexin':ti OR 'cephalosporanic acid':ti OR 'cephalosporanic acids':ti OR 'cephalosporin antibiotic':ti OR 'cephalosporin antibiotics':ti

OR 'cephalosporin':ti OR 'chlornitromycin':ti OR 'chlorocid':ti OR 'chloromycetin':ti OR 'ciprofloxacin':ti OR 'clarithromycin':ti OR 'cloranfenicol':ti OR 'dalbavancin':ti OR 'detreomycin':ti OR 'dicloxacillin':ti OR 'doxycycline':ti OR 'erythromycin':ti OR 'fidaxomicin':ti OR 'fluoroquinolone':ti OR 'fluoroquinolones':ti OR 'fucithalmic':ti OR 'furadantin':ti OR 'furadantine':ti OR 'furadoine':ti OR 'furadonine':ti OR 'furantoin':ti OR 'fusidate sodium':ti OR 'fusidic acid':ti OR 'fusidin':ti OR 'gentamicin':ti OR 'levofloxacin':ti OR 'levomycetin':ti OR 'macrodantin':ti OR 'macrolide':ti OR 'minocycline':ti OR 'moxifloxacin':ti OR 'nafcillin':ti OR 'ofloxacin':ti OR 'ophthochlor':ti OR 'oritavancin':ti OR 'oxacillin':ti OR 'penicillin g':ti OR 'penicillin v':ti OR 'penicillin':ti OR 'phenoxymethylpenicillin':ti OR 'proloprim':ti OR 'roxithromycin':ti OR 'sarecycline':ti OR 'silver fusidate':ti OR 'sodium fusidate':ti OR 'stanicide':ti OR 'sulfacetamide':ti OR 'sulfadiazine silver':ti OR 'sulfasalazine':ti OR 'syntomycin':ti OR 'telavancin':ti OR 'trimpex':ti OR 'vancocin hcl':ti OR 'vancocin':ti OR 'vancocine':ti OR 'vancomycin hydrochloride':ti OR 'vancomycin sulfate':ti OR 'vancomycin':ti) AND ('glomerulus filtration rate'/exp OR 'inulin clearance'/exp OR 'kidney function test'/exp OR 'renal clearance'/exp OR 'gfr':ti OR 'glomerular filtration rate':ti OR 'glomerular filtration rates':ti OR 'glomerular filtration rate'/exp OR 'inulin clearance':ti OR 'kidney function test':ti OR 'kidney function tests':ti OR 'kidney function':ti OR 'pharmacogenomic variants':ti OR 'pharmacogenomic variant':ti OR 'renal clearance':ti OR 'renal function':ti OR (('rate':ti OR 'clearance':ti) AND (metaboli*:ti OR pharmacokinetic*:ti)) OR 'clearance rate':ti OR 'clearance rates':ti) NOT ([animals]/lim NOT [humans]/lim)

Ovid Platform: EBM Reviews - Cochrane Clinical Answers, EBM Reviews - Cochrane Database of Systematic Reviews, EBM Reviews - Cochrane Central Register of Controlled Trials, and EBM Reviews - Cochrane Methodology Register

35 results on 16 August 2022

((exp "bumetanide"/ OR exp "ethacrynic acid"/ OR exp "furosemide"/ OR exp "sodium potassium chloride symporter inhibitors"/ OR exp "torsemide"/ OR (bumetanide OR bumethanide OR bumex OR burinex OR demadex OR edecrin OR errolon OR "etacrynic acid" OR "ethacrinic acid" OR "ethacrynate sodium" OR "ethacrynic acid" OR fordiuran OR frusemid OR frusemide OR furanthril OR furosemide OR fusid OR "high ceiling diuretics" OR hydromedin OR lasix OR "loop diuretic" OR "loop diuretics" OR "loop inhibitor" OR "loop inhibitors" OR piretanide OR "sodium potassium chloride symporter inhibitors" OR torasemide).ab,ti.) AND (exp "Anti-Bacterial Agents"/ OR exp "Anti-Infective Agents"/ OR exp "Fusidic acid"/ OR exp "Aminoglycosides"/ OR exp "Cephalosporins"/ OR exp "Chloramphenicol"/ OR exp "Fluoroquinolones"/ OR exp "Macrolides"/ OR exp "Nitrofurantoin"/ OR exp "Penicillins"/ OR exp "Tetracycline"/ OR exp "Trimethoprim"/ OR exp "Vancomycin"/ OR (aminoglycoside OR amoxicillin OR amphenicol OR amphenicols OR ampicillin OR "anti bacterial agent" OR "anti bacterial agents" OR "anti bacterial compound" OR "anti bacterial compounds" OR "anti mycobacterial agent" OR "anti mycobacterial agents" OR anti-bacteria OR "antibacterial agent" OR "anti-bacterial agent" OR "antibacterial agents" OR "anti-bacterial agents" OR "anti-bacterial compound" OR "anti-bacterial compounds" OR antibiotic OR anti-biotic OR antibiotics OR anti-biotics OR "antimycobacterial agent" OR "anti-mycobacterial agent" OR "antimycobacterial agents" OR "anti-mycobacterial agents" OR azithromycin OR "bacteriocidal agent" OR "bacteriocidal agents" OR bacteriocide OR bacteriocides OR beta-lactam OR cefaclor OR cefadroxil OR cefazolin OR cefixime OR cefoxitin OR ceftriaxone OR cefuroxime OR cephalexin OR "cephalosporanic acid" OR "cephalosporanic acids" OR "cephalosporin antibiotic" OR "cephalosporin antibiotics" OR cephalosporin OR chlornitromycin OR chlorocid OR chloromycetin OR ciprofloxacin OR clarithromycin OR cloranfenicol OR dalbavancin OR detreomycin OR dicloxacillin OR doxycycline OR erythromycin OR fidaxomicin OR fluoroquinolone OR fluoroquinolones OR fucithalmic OR furadantin OR furadantine OR furadoine OR furadonine OR furantoin OR "fusidate sodium" OR "fusidic acid" OR fusidin OR gentamicin OR levofloxacin OR levomycetin OR macrodantin OR macrolide OR minocycline OR moxifloxacin OR nafcillin OR ofloxacin OR ophthochlor OR oritavancin OR oxacillin OR "penicillin g" OR "penicillin v" OR penicillin OR phenoxymethylpenicillin OR proloprim OR roxithromycin OR sarecycline OR "silver fusidate" OR "sodium fusidate" OR stanicide OR sulfacetamide OR "sulfadiazine silver" OR sulfasalazine OR syntomycin OR telavancin OR trimpex OR "vancocin hcl" OR vancocin OR vancocine OR "vancomycin hydrochloride" OR "vancomycin sulfate" OR vancomycin).ab,ti) AND (exp "glomerular filtration rate"/ OR exp "metabolic clearance rate"/ OR exp "metabolism"/ OR exp "pharmacogenomics variants"/ OR exp "pharmacokinetics"/ OR (gfr OR "glomerular filtration rate" OR "glomerular filtration rates" OR "inulin clearance" OR "kidney function test" OR "kidney function tests" OR "kidney function" OR metabolism OR "pharmacogenomic variants" OR pharmacokinetics OR pharmacology OR "renal

clearance" OR "renal function" OR metaboli* OR pharmacokinetic* OR "clearance rate" OR "clearance rates" OR "drug clearance").ab,ti.)) NOT (exp animals/ NOT exp humans/)
