# Supplementary material for: Systematic Review and Meta-Analysis of the Effect of Loop Diuretics on Antibiotic Pharmacokinetics
Source: Pharmaceutics. 2023 May 5;15(5):1411. doi: 10.3390/pharmaceutics15051411 (PMC10224453; doi:10.3390/pharmaceutics15051411)
Supplement: Supplementary file 1 [file pharmaceutics-15-01411-s001.zip › Supplementary Tables S1 and S2.pdf]

Table S1: Subgroup analysis by antibiotic class

Parameter	Subgroup	N Studies	ROM	95% CI	I^2 (%)
AUC	Beta-Lactam	5	1.22	1.07 – 1.4	0
	Aminoglycoside	5	1.18	1.05 – 1.32	0
	Other	1	1.12	0.99 – 1.26	0
Half-life	Beta-Lactam	5	1.07	0.97 – 1.19	0
	Aminoglycoside	3	0.99	0.88 – 1.12	0
	Other	3	1.05	0.73 – 1.51	74.4
V_d	Beta-Lactam	3	0.84	0.73 – 0.96	20.9
	Aminoglycoside	3	0.9	0.77 – 1.05	0
	Other	1	1	0.82 – 1.21	0
C_{max}	Beta-Lactam	3	1.28	1.07 – 1.53	0
	Aminoglycoside	1	1.03	0.86 – 1.23	0
	Other	1	1.14	0.97 – 1.35	0
eGFR	Beta-Lactam	6	0.98	0.79 – 1.22	89
	Aminoglycoside	4	0.87	0.64 – 1.18	81.5
	Other	1	1.11	0.99 – 1.26	0
A_e	Beta-Lactam	6	1.37	1.11 – 1.69	80.2
	Aminoglycoside	3	1.37	0.85 – 2.21	94
	Other	2	1.03	0.5 – 2.13	82.3

Table S2: Subgroup analysis by diuretic

Parameter	Subgroup	N Studies	ROM	95% CI	I^2 (%)
AUC	Furosemide	9	1.19	1.09 – 1.28	0
	Other Diuretic	2	1.11	0.95 – 1.28	0
Half-life	Furosemide	8	1.07	1 – 1.15	22.9
	Other Diuretic	3	1	0.89 – 1.14	32.7
V_d	Furosemide	4	0.84	0.75 – 0.94	0
	Other Diuretic	3	0.94	0.83 – 1.08	0
C_{max}	Furosemide	4	1.21	1.07 – 1.36	0
	Other Diuretic	1	1.03	0.86 – 1.23	0
eGFR	Furosemide	8	0.94	0.77 – 1.15	87.6
	Other Diuretic	3	0.99	0.76 – 1.29	84.6
A_e	Furosemide	7	1.24	0.97 – 1.59	84.8
	Other Diuretic	4	1.4	0.98 – 2.01	95.4
